# Evaluating the impact of early dignity therapy on quality of life in patients with brain tumors: A pilot study

**DOI:** 10.1017/S1478951525100217

**Published:** 2025-08-22

**Authors:** Aaron Palachi, Janet Ellis, Mahiya Habib, Claire Moroney, Elie Isenberg-Grzeda, Margaret Fitch, Mary Jane Esplen, Arjun Saghal, Melissa B. Korman

**Affiliations:** 1 Sunnybrook Health Sciences Centre, Toronto, ON, Canada; 2 Department of Psychology, Toronto Metropolitan University, Toronto, ON, Canada; 3 Temerty Faculty of Medicine, University of Toronto, Toronto, ON, Canada

**Keywords:** Dignity therapy, brain cancer, early intervention, palliative care, psychosocial well-being

## Abstract

**Objectives:**

Brain tumors are associated with negative changes in sense of self and increased distress early in the illness trajectory. Dignity Therapy (DT) is a brief 2-session therapeutic intervention for patients at end-of-life (EOL) that helps conserve a patient’s sense of dignity or self. DT has shown positive results for patients at EOL including increased meaning, improved quality of life (QOL), and reduced distress, with limited research to date on patients early in their illness trajectory (non-EOL). This pre-post design pilot study investigated the benefits and feasibility of DT for 2 groups of patients with incurable brain tumors.

**Methods:**

A total of 51 participants were recruited, of whom 39 participated. Participants were grouped as EOL (prognosis < 1 year, *n* = 21) and non-EOL (prognosis > 1 year, *n* = 18). Participants completed self-report measures to determine changes in QOL, psychosocial well-being (i.e., spiritual well-being, connection, and posttraumatic growth), and death anxiety, at baseline, 1 week, and 5 weeks post-intervention.

**Results:**

The intervention had a high completion rate, with 37 of 39 participants (95%) completing DT. Linear regression models fitted with generalized estimating equations (GEEs) showed within- and between-group significant changes in all domains for both groups, but were particularly beneficial for non-EOL participants.

**Significance of results:**

This study demonstrated that DT effectively enhanced psychosocial well-being in patients with brain tumors, including reductions in death anxiety and dignity-related distress. Non-EOL participants benefited most and had higher completion rates, highlighting the intervention’s feasibility and the need for further research in earlier stages of terminal illness.

## Introduction

Irreversible illness, such as terminal brain cancer, can decrease a person’s quality of life (QOL) through loss of autonomy, social, functional, and existential challenges, and decreased dignity (Korman et al. 2021; Pertz et al. 2022). In fact, receiving a terminal diagnosis is a potentially traumatic event (Davidson et al. 2023; Dimitrov et al. 2019). Brain tumors are often associated with changes in sense of self earlier in the illness trajectory compared to other cancers (Rimmer et al. 2023); patients may experience loss of vital abilities, impaired cognition or coordination, and seizures, potentially impacting independence (Chieffo et al. 2023).

Most people with brain tumors experience ongoing distress affecting their well-being (Fehrenbach et al. 2021; Halkett et al. 2022), including existential (i.e., feeling demoralized, hopeless, questioning meaning, life, and death), emotional (i.e., sadness, fear, worry, anger, and guilt), physical, functional (i.e., ability to carry out usual activities), and symptom-related distress (Korman et al. 2021). Such distress may contribute to the onset of depressive and anxiety-related disorders, death anxiety, negative end-of-life (EOL) experiences, and requests for hastened death (Haywood et al. 2024; Seiler et al. 2024; Sinnarajah et al. 2022). Therapeutic interventions aiding individuals near EOL that focus on helping patients adjust to their diagnosis, symptoms, and distress can foster positive psychosocial outcomes and improve QOL (Rodin et al. 2020; Warth et al. 2019). When caring for the psychosocial needs of people with incurable disease, the goal is to support dignity and meaning in life and death, while reminding people of who they are beyond their illness (Zheng et al. 2023).

### Dignity therapy

Dignity Therapy (DT), created by Harvey Chochinov, is a 2-session therapeutic intervention for patients at EOL to reduce distress and facilitate meaning-making (Chochinov et al. 2005). Patients are encouraged to reflect on their lives, identify what is most significant to them, and most important in their remembrance and legacy (Chochinov et al. 2005). DT sessions are used to develop a “Legacy Document,” a memoir for patients’ loved ones, which can give the patient a sense of continuity. DT studies found high patient satisfaction, increased dignity and connection with others, and decreased desire for hastened death (Chochinov et al. 2011; Emanuel et al. 2023; Korman et al. 2021; Nunziante et al. 2021; Seiler et al. 2024). However, there are mixed findings in how DT affects QOL, spiritual, and psychosocial well-being, suggesting heterogeneity in its effects, a need for longer follow-ups, or earlier intervention (Seiler et al. 2024; Xiao et al. 2019; Zhang et al. 2022).

While DT is recommended for patients near EOL, recent literature suggests that patients with terminal cancer may benefit from DT earlier on due to unique symptoms contributing to loss of independence, diminished sense of self, and cognitive impairment earlier in their illness trajectory (Nunziante et al. 2021; Zhang et al. 2022). Despite high rates of distress experienced by terminally ill patients with brain tumors, research is lacking on testing the efficacy of brief, feasible interventions such as DT to address psychosocial distress and improve QOL early in the illness trajectory.

### Present study

This pilot study aimed to address these gaps and assess the impact of DT on QOL and psychosocial well-being (connection, psychosocial and spiritual well-being, death anxiety, and posttraumatic growth) for patients both at EOL and those not at EOL (non-EOL). This study explored whether these domains of QOL and psychosocial well-being, particularly death anxiety, are modifiable in this population, and if implementing DT with non-EOL patients is feasible. This study had 2 hypotheses: (1) both EOL and non-EOL patients who receive DT would experience overall improvement in measures of QOL, psychosocial well-being, and death anxiety; (2) there would be no significant differences between groups on primary measures of QOL, psychological well-being and death anxiety at all time points suggesting similar benefits of DT for both groups.

## Methods

### Design

This study used a 2-group pre-posttest design to pilot methods for future studies. No randomization or control groups were used. This study was approved by the Research Ethics Board (REB#2467) at Sunnybrook Health Sciences Centre.

### Participants

Participants were recruited from the Odette Cancer Centre in Toronto, Ontario, through flyers and oncologist referrals. Inclusion criteria were as follows: (1) aged 18–80 years, (2) undergoing treatment for an incurable brain tumor (i.e., cannot be eradicated by medical treatment), (3) English speaking, (4) able to provide consent, and (5) able to complete the intervention and study measures (not limited by cognitive impairment or physical deterioration). Participants were placed into 2 groups: (1) EOL: prognosis of less than 1 year to live and (2) non-EOL: prognosis of 1–10 years to live as deemed by expert medical opinion. Participants’ demographics (i.e., age, gender, diagnosis, and prognosis) were collected at baseline.

### Intervention

The intervention was conducted according to the DT protocol, with 2 90-minute sessions (additional sessions available if necessary; Chochinov et al. 2005). Participants received DT with a nurse practitioner or a clinical research coordinator (referred to as “DT therapists”), supervised by the PI (a psychiatrist), all of whom completed the 3-day DT training facilitated by Chochinov. The PI performed fidelity checks on recorded sessions and confirmed all sessions adhered to the protocol (Appendix A). In session 1, participants were asked open-ended questions developed for DT (Chochinov et al. 2005). Sessions were audio-recorded and transcribed by a research assistant and edited by DT therapists to create a “Legacy Document,” which was read to and co-edited with the participant. After final edits, participants could obtain copies of the “Legacy Document” to share with loved ones.

### Data collection

Research assistants collected self-report questionnaires prior to the pandemic; thereafter, questionnaires were collected via LimeSurvey from consenting participants. Questionnaires were completed at baseline prior to the first DT session, 1 week post-intervention (T1), and 5 weeks post-intervention (T2). Data collection occurred between November 2019 and September 2022.

### Primary outcomes and measures

*QOL* was measured using the Functional Assessment in Cancer Therapy for Patients with Brain Cancer (FACT-Br; Weitzner et al. 1995), a scale that measures QOL for patients with primary brain tumors on separate subscales (emotional distress, social connectedness and perceived support, and functional and physical well-being). Using a 5-point Likert scale, item scores range from 0 (not at all) to 4 (very much), with higher scores indicating better QOL. The brain cancer subscale measured illness-related symptoms and concerns, with lower scores indicating greater impairment or symptom burden. Internal consistency was good-excellent at baseline, T1, and T2 (α = .86–.91; α = .81–.91; α = .74–.93, respectively).

*Death anxiety* was evaluated using the Death and Dying Distress Scale (DADDS; Lo et al. 2011). This 15-item scale measures thoughts and feelings related to life, death, and dying that are present over the last 2 weeks for patients with advanced cancer. Using a 6-point Likert scale, responses range from 0 (no distress) to 5 (extreme distress). Scores range between 0 and 75, suggesting mild (0–25), moderate (26–50), and severe (51–75) death anxiety. Internal consistency was excellent at baseline, T1, and T2 (α = .96; α = .97; α = .96 respectively).

### Secondary outcomes and measures

*Connection with others* was determined using the University of California, Los Angeles (UCLA) 3-item Loneliness Scale (Russell 1996), which measures 3 dimensions of loneliness (relational connectedness, social connectedness, and self-perceived isolation) on a 3-point Likert scale. Item scores range from 1 (hardly ever) to 3 (often), with total scores from 3 to 9; scores above 6 suggest loneliness. Internal consistency at baseline, T1, and T2 was deemed good (α = .81; α = .79; α = .75, respectively).

*Spiritual well-being* was measured using the Spiritual Well-being Scale (FACIT-Sp-12; Peterman et al. 2002). Using a 6-point Likert scale, the FACIT-Sp-12 determines perception of spiritual QOL across 3 subscales (meaning, peace, and faith) with responses ranging from 1 (strongly disagree) to 6 (strongly agree). Scores are presented as low (20–40), moderate (41–99), and high (100–120) spiritual well-being. Internal consistency was good across time points (baseline, α = .86–.88; T1, α = .76–.89; T2, α = .81–.93).

*Dignity* was measured using the Patient Dignity Inventory (PDI; Chochinov et al. 2008). This 25-item 5-point Likert scale measures dignity-related distress in patients across 5 subscales (i.e., symptom, existential, dependency, peace of mind, and social support distress) reported over the past few days with scores ranging from 1 (not a problem) to 5 (an overwhelming problem). Internal consistency across all time points was considered good-excellent (baseline, α = .90; T1, α = .85; T2, α = .91).

*Posttraumatic growth* was measured using the Post Traumatic Growth Inventory (PTGI; Tedeschi and Calhoun 1996). This 21-item survey measures PTG and self-improvement across 5 domains (personal strength, new possibilities, improved relationships, spiritual growth, and appreciation for life) using a 6-point Likert scale ranging from 0 (I did not experience this as a result of my crisis) to 5 (I experienced this change to a very great degree as a result of my crisis). Scores are interpreted as 0–45 (none-low PTG) and 46+ (medium-high PTG). Internal consistency was deemed excellent across baseline, T1, and T2 (α = .94, α = .96, and α = .97 respectively).

### Feasibility outcomes

Data regarding participant uptake, and intervention and data completion were gathered to determine the feasibility of conducting future trials (Pearson et al. 2020). Feasibility data included average length of each DT session, number of sessions conducted with each participant, and participant attrition across the study.

### Statistical analyses

Descriptive statistics, including means and standard deviations, were used to measure change in QOL and psychosocial well-being on all outcomes across time points for each group (Hypothesis 1). Statistical analyses were conducted using R statistical software (RStudio Team 2020). Linear regression models fitted with GEEs were used to explore changes in QOL and psychosocial well-being. Cross-sectional comparisons between both groups were conducted to determine within- and between-group differences across all time points (Hypothesis 2).

## Results

### Sample characteristics

A total of 51 participants were recruited for the study, with a final sample of 39 (*n* = 21 EOL group; *n* = 18 non-EOL group). There were 25 women (*n* = 13 EOL; *n* = 12 non-EOL) and 14 men (*n* = 8 EOL; *n* = 6 non-EOL) with an overall mean age of 58 years (see Table 1). Of all participants recruited, 37 completed all sessions of DT. In terms of brain tumor diagnoses, metastases were the most common (*n* = 13) in the EOL group, and malignant neoplasm of the brain (*n =* 6) was the most common for non-EOL participants. Mean prognosis for each group was ∼9.5 months (EOL) and ∼5.5 years (non-EOL).

**Table 1.** Participant demographic information

	EOL (*n =* 21)		Non-EOL (*n =* 18)	
Demographic variable	*N*	% of group	*N*	% of group
Age (range: 22–79)	*M =* 62	SD *=* 10	*M =* 54	SD *=* 14
Gender identity				
Man	8	38.1	6	33.3
Woman	13	61.9	12	66.7
Brain tumor type				
Metastases	13	61.9	6	33.3
Glioblastoma	5	23.8	1	5.6
Meningioma	3	14.3	1	5.6
Chordoma	–	–	1	5.6
Malignant neoplasm of the brain	–	–	6	33.3
Other	–	–	3	16.7
Prognosis				
3–6 months	4	19.0	–	–
6 months–1 year	7	33.3	–	–
1 year	9	42.9	–	–
1–2 years	–	–	1	5.6
3–5 years	–	–	3	16.7
5 years	–	–	4	22.2
5–10 years	–	–	8	44.4

*Note*: Participant demographic information was collected at baseline. One EOL participant’s chart did not specify their exact prognosis but was identified as less than 1 year. Two non-EOL participants’ charts did not specify their exact prognosis but were identified as more than 1 year.

### Primary outcomes

#### Quality of life

For the EOL group, mean FACT-Br scores indicated significant changes throughout the study (Table 2). There was a significant increase in functional well-being from baseline to T1 (β = 1.35, SE = 0.62, *p =* .029), followed by significant decreases in functional (β = −3.00, SE = 1.27, *p =* .018) and physical (β = −2.19, SE = 0.89, *p =* .014) well-being from T1 to T2. From baseline to T2, EOL participants’ scores on the Brain Cancer subscale significantly decreased (β = −7.47, SE = 2.72, *p =* .006), indicating greater illness-related impairment or burden. Non-EOL participants only had significant increases in scores of emotional well-being, and only between baseline and T1 (β = 2.39, SE = 0.78, *p =* .002). Compared to the non-EOL group, EOL participants had significantly lower emotional well-being scores at all time points, lower functional well-being scores at baseline and T2, and greater impairment on the Brain Cancer subscale at T1 and T2 (Table 3).

**Table 2.** Descriptive statistics and within-group differences on all measures across timepoints for both groups

	Baseline		T1		T2	
Measure	EOL (*n* = 21)	Non-EOL (*n* = 18)	EOL (*n* = 17)	Non-EOL (*n* = 16)	EOL (*n* = 15)	Non-EOL (*n* = 13)
**FACT-Br (SD)**						
Physical WB^a^	20.05 (6.53)	21.26 (5.97)	22.43 (4.09)	22.00 (6.28)	20.33 (5.91)^b^^,^*	20.93 (7.70)
Social WB^a^	19.41 (4.88)	17.89 (6.00)	19.64 (4.91)	18.79 (4.25)	18.47 (4.76)	18.33 (4.01)
Emotional WB^a^	13.50 (5.65)	17.21 (5.11)	15.57 (5.37)	20.07 (3.08)^c^^,^*	14.13 (6.09)	18.73 (5.35)
Functional WB^a^	13.55 (6.38)	18.42 (6.72)	16.21 (7.51)^c^^,^*	19.00 (6.18)	12.47 (6.72)^b^^,^*	19.40 (6.22)
Brain cancer	62.36 (14.76)	70.16 (17.00)	63.15 (12.07)	70.43 (13.50)	56.80 (17.73)^d^^,^**	71.80 (15.37)
**DADDS (SD)**	30.50 (19.38)	19.00 (19.70)	23.93 (22.32)	11.33 (10.30)^c^^,^*	27.27 (20.87)	15.71 (13.80)
**UCLA-3 (SD)**	4.68 (1.55)	4.60 (1.96)	3.86 (1.51)^c^^,^**	3.73 (1.49)	4.93 (1.53)^b^^,^**	4.57 (1.70)
**FACIT-Sp-12 (SD)**	32.09 (9.66)	30.68 (11.75)	33.08 (10.44)	35.79 (9.51)	31.20 (12.36)	34.53 (12.47)
Meaning	13.23 (2.78)	12.32 (4.11)	13.23 (3.24)	14.21 (2.39)	12.20 (3.71)^d^^,^*	13.40 (3.62)
Peace	9.82 (4.09)	7.79 (5.66)	9.77 (4.66)	9.86 (5.57)	9.33 (5.00)	9.33 (5.35)
Faith	10.23 (3.95)	8.15 (5.44)	9.36 (4.96)	9.47 (5.94)	9.80 (4.72)	9.93 (4.83)
**PDI (SD)**	49.68 (15.66)	41.45 (16.31)	44.36 (16.50)	34.33 (8.08)^c^^,^*	47.53 (21.18)	35.00 (11.92)
Existential distress	12.77 (5.10)	10.55 (5.23)	10.93 (5.05)	8.53 (2.77)^c^^,^*	12.00 (6.74)	8.13 (2.83)^d^^,^*
Symptom distress	14.00 (4.65)	11.75 (5.05)	11.86 (4.38)^c^^,^*	9.60 (2.69)^c^^,^*	12.87 (5.33)	10.40 (4.61)
Social support distress	3.86 (1.36)	4.10 (1.74)	4.21 (2.36)	3.27 (0.70)^c^^,^**	3.50 (1.09)	3.33 (0.62)^d^^,^*
Dependency distress	4.55 (2.32)	3.50 (1.00)	4.07 (1.33)	3.60 (0.91)	4.93 (2.91)	3.40 (0.74)
Peace of mind distress	5.18 (1.50)	4.65 (1.69)	5.21 (2.19)	3.72 (1.28)^c^^,^**	4.80 (1.97)	3.80 (1.57)^d^^,^*
**PTGI (SD)**	42.55 (21.69)	46.00 (30.62)	45.21 (23.79)	48.20 (33.68)	36.73 (21.06)	56.07 (35.42)

a Represents well-being.

b Indicates a statistically significant change from T1 to T2.

c Indicates a statistically significant change from baseline to T1.

d Indicates a statistically significant change from baseline to T2.

* *p* < .05, **p < .01.

**Table 3.** GEE linear regression analyses comparing groups at each timepoint

Measure	β	SE	95% CI	*p*
**FACT-Br (Physical WB^a^)**				
Baseline × Group (non-EOL × EOL)	−1.39	1.90	[−5.11, 2.33]	.465
T1 × Group (non-EOL × EOL)	−1.27	1.89	[−4.97, 2.43]	.502
T2 × Group (non-EOL × EOL)	−0.60	2.42	[−5.35, 4.15]	.804
**FACT-Br (Social WB^a^)**				
Baseline × Group (non-EOL × EOL)	1.75	1.71	[−1.61, 5.11]	.306
T1 × Group (non-EOL × EOL)	1.48	1.42	[−1.30, 4.25]	.298
T2 × Group (non-EOL × EOL)	0.13	1.55	[−2.91, 3.18]	.931
**FACT-Br (Emotional WB^a^)**				
Baseline × Group (non-EOL × EOL)	−3.71	1.61	[−6.87, −0.55]	.021^*^
T1 × Group (non-EOL × EOL)	−5.12	1.49	[−8.04, −2.20]	<.001^***^
T2 × Group (non-EOL × EOL)	−4.60	2.02	[−8.56, −0.64]	023*
**FACT-Br (Functional WB^a^)**				
Baseline × Group (non-EOL × EOL)	−5.05	1.94	[−8.86, −1.24]	.009**
T1 × Group (non-EOL × EOL)	−3.66	2.00	[−7.58, 0.26]	.067
T2 × Group (non-EOL × EOL)	−6.93	2.28	[−11.41, −2.56]	.002**
**FACT-Br (Brain cancer)**				
Baseline × Group (non-EOL × EOL)	−15.77	9.81	[−35.01, 3.47]	.108
T1 × Group (non-EOL × EOL)	−21.15	9.26	[−39.30,−3.00]	.022*
T2 × Group (non-EOL × EOL)	−15.00	5.85	[−26.47,−3.53]	.010*
**DADDS**				
Baseline × Group (non-EOL × EOL)	11.47	5.82	[0.06, 22.88]	.049*
T1 × Group (non-EOL × EOL)	12.45	5.86	[0.96, 23.94]	.034*
T2 × Group (non-EOL × EOL)	11.55	6.30	[−0.80, 23.91]	.067
**UCLA-3**				
Baseline × Group (non-EOL × EOL)	0.08	0.55	[−1.00, 1.57]	.884
T1 × Group (non-EOL × EOL)	0.19	0.52	[−0.84, 1.22]	.718
T2 × Group (non-EOL × EOL)	0.36	0.58	[−0.78, 1.50]	.533
**FACIT-Sp-12 (Total)**				
Baseline × Group (non-EOL × EOL)	1.31	3.33	[−5.21, 7.83]	.694
T1 × Group (non-EOL × EOL)	−0.34	3.08	[−6.37, 5.69]	.912
T2 × Group (non-EOL × EOL)	−3.33	4.38	[−11.92, 5.25]	.447
**FACIT-Sp-12 (Meaning)**				
Baseline × Group (non-EOL × EOL)	0.88	1.09	[−1.25, 3.02]	.417
T1 × Group (non-EOL × EOL)	−0.31	0.93	[−2.14, 1.52]	.739
T2 × Group (non-EOL × EOL)	−1.20	1.29	[−3.73, 1.33]	.353
**FACIT-Sp-12 (Peace)**				
Baseline × Group (non-EOL × EOL)	2.04	1.56	[−1.03, 5.10]	.193
T1 × Group (non-EOL × EOL)	1.48	1.60	[−1.67, 4.62]	.357
T2 × Group (non-EOL × EOL)	0.00	1.83	[−3.58, 3.58]	1.00
**FACIT-Sp-12 (Faith)**				
Baseline × Group (non-EOL × EOL)	2.28	1.48	[−0.61, 5.17]	.123
T1 × Group (non-EOL × EOL)	0.84	1.79	[−2.67, 4.36]	.638
T2 × Group (non-EOL × EOL)	−0.13	1.69	[−3.44, 3.17]	.937
**PDI (Total)**				
Baseline × Group (non-EOL × EOL)	8.65	4.77	[−0.69, 18.00]	.070
T1 × Group (non-EOL × EOL)	11.05	4.45	[2.34, 19.77]	.013*
T2 × Group (non-EOL × EOL)	12.53	6.06	[0.65, 24.42]	.039*
**PDI (Existential distress)**				
Baseline × Group (non-EOL × EOL)	2.37	1.56	[−0.68, 5.43]	.128
T1 × Group (non-EOL × EOL)	2.99	1.40	[0.26, 5.73]	.032*
T2 × Group (non-EOL × EOL)	3.87	1.87	[0.20, 7.54]	.039*
**PDI (Symptom distress)**				
Baseline × Group (non-EOL × EOL)	2.58	1.46	[−0.29, 5.46]	.078
T1 × Group (non-EOL × EOL)	2.50	1.26	[0.04, 4.96]	.046*
T2 × Group (non-EOL × EOL)	2.47	1.76	[−0.98, 5.91]	.161
**PDI (Social support distress)**				
Baseline × Group (non-EOL × EOL)	−0.23	0.48	[−1.17, 0.70]	.623
T1 × Group (non-EOL × EOL)	0.81	0.58	[−0.33, 1.96]	.162
T2 × Group (non-EOL × EOL)	0.17	0.32	[−0.46, 0.80]	.602
**PDI (Dependency distress)**				
Baseline × Group (non-EOL × EOL)	1.05	0.53	[0.00, 2.09]	.049*
T1 × Group (non-EOL × EOL)	0.70	0.40	[−0.09, 1.50]	.081
T2 × Group (non-EOL × EOL)	1.53	0.75	[0.06, 3.00]	.041*
**PDI (Peace of mind distress)**				
Baseline × Group (non-EOL × EOL)	0.49	0.49	[−0.47, 1.44]	.317
T1 × Group (non-EOL × EOL)	1.39	0.57	[−0.27, 2.52]	.015*
T2 × Group (non-EOL × EOL)	1.00	0.63	[−0.23, 2.23]	.111
**PTGI**				
Baseline × Group (non-EOL × EOL)	−2.68	7.93	[−18.22, 12.86]	.736
T1 × Group (non-EOL × EOL)	−4.16	9.84	[−23.45, 15.12]	.672
T2 × Group (non-EOL × EOL)	−19.33	10.28	[−39.48, 0.82]	.060

a *Note*: Refers to well-being. The non-EOL group is the group reference point.

* *p* < .05, **p < .01, ***p < .001.

#### Death anxiety

Mean scores on the DADDS significantly decreased for non-EOL participants between baseline and T1 (β = −6.51, SE = 3.31, *p =* .049; Table 2). While EOL participants’ death anxiety scores decreased from baseline to T1, it was not significant. Both groups had increased death anxiety from T1 to T2, but it was not statistically significant. Mean scores for both groups within our sample indicate that mild death anxiety was present for the non-EOL group, and moderate death anxiety was present for the EOL group across the study. Compared to the non-EOL group (Table 3), participants at EOL scored significantly higher on death anxiety at baseline and T1, but not T2.

### Secondary outcomes

#### Connection with others

Participants in the EOL group had a significant reduction in UCLA-3 scores from baseline to T1 (β = −0.72, SE = 0.25, *p =* .005), followed by a significant increase from T1 to T2 (β = 0.95, SE = 0.34, *p =* .006; Table 2). Non-EOL participants, while following a similar trajectory, had no significant changes. Based on cut-off scores, neither group’s mean scores suggested the presence of loneliness within the sample, and no significant group differences were observed (Table 3).

#### Spiritual well-being

Changes in total FACIT-Sp-12 scores across all time points were not significant for either group (Table 2). Mean scores for both groups at each time point suggest low spiritual well-being. However, meaning subscale scores for EOL participants were significantly lower from baseline to T2 (β = −0.96, SE = 0.47, *p =* .043), indicating less meaning closer to EOL. While scores on the meaning subscale fluctuated across time points for the non-EOL group, these changes were not statistically significant. There were no significant differences between groups at each time point for any measure of spiritual well-being (Table 3). Finally, when considering the change of time point between groups, there was a significant decrease in scores for the EOL group compared to the non-EOL group on the meaning subscale from baseline to T2 (Appendix B).

#### Dignity

Mean PDI scores showed significant changes across time points, mainly for the non-EOL group (Table 2). From baseline to T1, non-EOL participants had significant reductions in total scores (β = −6.05, SE = 2.50, *p =* .015) and all domains of dignity-related distress except dependency distress: existential (β = −1.83, SE = 0.87, *p =* .036), symptom (β = −1.79, SE = 0.77, *p =* .020), social support (β = −0.75, SE = 0.25, *p =* .003), and peace of mind distress (β = −0.92, SE = 0.28, *p =* .001). From baseline to T2, non-EOL participants had significant further reductions in existential (β = −1.85, SE = 0.82, *p =* .024), social support (β = −0.65, SE = 0.26, *p =* .011), and peace of mind (β = −0.90, SE = 0.40, *p =* .024) distress. EOL participants only had significant decreases in symptom distress between baseline and T1 (β = 1.78, SE = 0.72, *p =* .013). Overall, all study participants had significant decreases in scores on the total PDI, and symptom, social, and peace of mind distress domains between baseline and T1, and only social support and peace of mind domains from baseline to T2 (Appendix B). Analysis of the interaction between time point and group showed that the EOL group had a significantly higher change in social support distress than the non-EOL group from baseline to T1 (Appendix B). At baseline, the EOL group scored significantly higher on dependency distress than the non-EOL group (Table 3). Additionally, at T1, the EOL group had significantly greater total, existential, symptom, and peace of mind distress, and at T2, significantly higher total, existential, and dependency distress compared to the non-EOL group.

#### Posttraumatic growth

Neither EOL nor non-EOL groups had significant changes in mean PTGI scores across the study (Table 2). While not significant, mean PTG scores for the EOL group increased from baseline to T1, and then decreased from T1 to T2, indicating none-low PTG in this group. However, for the non-EOL group, mean scores continuously increased across all time points, but not significantly, maintaining medium-high PTG. No significant between-group differences were observed (Table 3).

### Intervention feasibility

**Figure 1.**
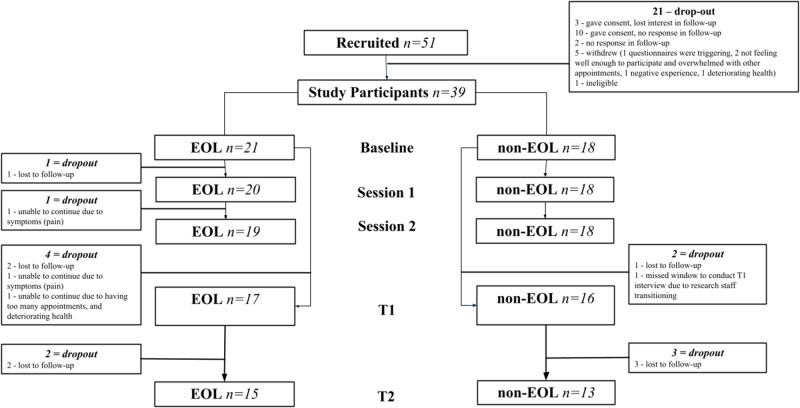
Study Recruitment, Enrolment and Intervention Completion for Both Groups.

Between recruitment and enrollment, 77% of potential participants were enrolled in the study (Figure 1). Completion rates of DT were 100% for non-EOL participants and 90% for EOL participants, with reasons for attrition reported in Figure 1. For completed measures, 72% of non-EOL and 71% of EOL participants were able to complete all study measures.

Session 1 of DT lasted 1.6 hours on average (range: 45 minutes–2 hours 45 minutes), with session 2 averaging 1 hour (range: 30 minutes–1 hour 46 minutes). Eight participants required a third session (*M* = 1 hour 13 minutes), one required a fourth session (50 minutes), and one required 4 additional sessions (range: 42 minutes–1 hour 46 minutes per session). The average overall time DT therapists spent with participants to complete the intervention was 2 hours 29 minutes.

## Discussion

This pre-post pilot study aimed to determine if DT was related to positive change in QOL, psychosocial well-being, and death anxiety in samples of non-EOL and EOL patients with brain tumors. This study explored differences in benefits for both groups and demonstrated the effectiveness and feasibility of conducting DT earlier in the illness trajectory, a future direction recommended by recent literature (Nunziante et al. 2021; Zhang et al. 2022).

Both groups experienced significant improvements in QOL, psychosocial well-being, and death anxiety, partially supporting Hypothesis 1. Non-EOL participants showed significant emotional well-being improvements from baseline to T1, indicating DT’s early benefits for coping with emotional responses compared to EOL participants. EOL participants showed significantly higher death anxiety prior to starting DT, indicating the need for interventions such as DT. While Emanuel et al. (2023) noted mixed effects of DT on death anxiety in EOL participants, our study revealed significant reductions in death anxiety for non-EOL participants immediately post-intervention (T1). Though death anxiety may be lower earlier in the illness trajectory, ongoing support may help these patients better adjust to their illness.

For connection with others and spiritual well-being, DT was significantly impactful for EOL participants from T1 to T2. This might indicate that while at first these individuals may have felt disconnected due to their progressive-palliative status, DT may have aided in fostering adjustment, acceptance, or connection with loved ones. A prior study noted not having enough time between post-intervention and follow-up (Karimi et al. 2020); this study’s longer follow-up period may have captured this positive change. Overall spiritual well-being scores did not significantly change for either group; however, EOL participants experienced significant reductions and difficulty with spiritual meaning. As found in prior literature, individuals at EOL may struggle with finding meaning, suggesting that current DT protocols may not adequately aid EOL patients in aspects of spiritual meaning (Iani et al. 2020). PTG scores showed moderate growth over time, with EOL participants’ gains waning and non-EOL participants’ gains increasing. However, PTG is a longer, heterogeneous process requiring longer follow-up to assess significant effects (Almeida et al. 2022).

Although DT was initially developed to foster dignity for those at EOL, this study revealed non-significant effects on dignity for EOL participants, similar to prior studies (Iani et al. 2020; Kelly et al. 2023; Nunziante et al. 2021; Seiler et al. 2024). Interestingly, several significant reductions in dignity-related distress were reported for non-EOL participants. There were immediate improvements in dignity on all domains except dependency distress from baseline to T1, and overall significant improvements on peace of mind, existential, and social support-related distress throughout the study. These findings suggest that implementing DT earlier may be particularly impactful for patients with a terminal illness, as perceptions of dignity may be more malleable during earlier stages.

This study hypothesized that DT would be as beneficial for non-EOL participants as EOL participants. Results showed significantly different changes in scores between both groups, failing to support Hypothesis 2, not because DT was not impactful for non-EOL participants, but the significant main effect suggests that DT was more beneficial for non-EOL participants than EOL participants. These differences may reflect either the advanced illness of EOL participants or the greater immediate psychosocial benefits of early DT for non-EOL participants.

Findings related to both feasibility and effectiveness in this pilot study support moving forward with a larger, randomized trial. Average time spent with patients was comparable to previous studies (Kelly et al. 2023; Labuschagne et al. 2024); most participants were retained throughout the intervention and data collection periods with perfect intervention completion in non-EOL participants (Labuschagne et al. 2024; Nunziante et al. 2021). Further, this study underscores the significance of introducing DT earlier in the illness trajectory to support adjustment to terminal illness.

### Clinical implications

This study has important implications for all patients who have received a diagnosis of, or are coping with, terminal illness. As receiving a terminal diagnosis may be traumatic for individuals (Davidson et al. 2023), implementing interventions such as DT to foster dignity and alleviate distress may aid in long-term psychosocial well-being and improve QOL (Lee and Jeong 2023; Zhang et al. 2022). Importantly, results showed that some of the positive effects of the intervention may taper off as patients’ illnesses progressed. Prior literature notes that an extended protocol for EOL patients may not be feasible; however, this study suggests that it should be considered for non-EOL patients (Seiler et al. 2024). Incorporating additional therapeutic models (e.g., Acceptance and Commitment Therapy) into DT for individuals at non-EOL may aid in extending the intervention beyond 2 sessions, bolstering acceptance with their diagnosis, and increasing dignity (Park 2023). Notably, physical and functional well-being indicators demonstrate a need to address physical symptoms of cancer in treatment, as these may be unchangeable, progressive, and contribute to decreased overall well-being.

### Study limitations and future directions

This study acknowledges several limitations. First, death anxiety yielded high standard deviations indicating heterogeneity in EOL experiences (Li et al. 2024). Second, some EOL participants found the measures difficult to complete due to either rapidly deteriorating health conditions or lack of time or energy. Next, longer follow-up times may have more strongly indicated sustained change post-intervention. Similarly, due to varying diagnoses within the sample, differences in symptom experience, severity, and impact may have contributed to variability in psychosocial well-being and QOL indices for participants (Iani et al. 2020; Seiler et al. 2024). Additionally, due to the inclusion criteria, these results are limited to patients without severe cognitive or physical impairments from brain tumors. Finally, the COVID-19 pandemic impacted research processes, with reduced volunteer support, and the need to transition to remote recruitment and data collection, which impacted attrition resulting in the ability to collect total data from only 15 participants per group.

Our findings reinforce the need for further research on DT for non-EOL patients. Future research should continue to investigate the impact of DT for non-EOL and EOL patients with randomized control study designs to evaluate the efficacy of DT compared to other brief palliative therapies (e.g., CALM therapy; Rodin et al. 2018). Mixed methods research can highlight relationships between DT and functional QOL to provide further insight as to how DT helps to remind patients of who they are beyond their illness. Culture may be an important piece in maintaining spiritual well-being, meaning, dignity, and other psychosocial well-being factors (Zhang et al. 2022), and insights from qualitative data could help develop a culturally informed framework for DT. Future studies may also include family or caregivers within their analysis to determine the effects of DT and the Legacy Documents on the patients’ support networks.

## Conclusions

Findings from this study support that DT is a valuable intervention for patients with brain tumors, enhancing psychosocial well-being and addressing key areas including death anxiety, dignity, and meaning among participants. The main finding demonstrated that DT was particularly beneficial for non-EOL participants, mitigating dignity-related distress and death anxiety compared to EOL participants, presenting novel insights as DT had yet to be explored in non-EOL patients. Completion rates and attrition suggest that DT is feasible for both groups, with higher completion and data collection rates with non-EOL participants, warranting further research in earlier stages of terminal illness. In measures of QOL and psychosocial well-being, results highlight heterogeneity in the experience of terminal cancer, emphasizing the need for person-centered, tailored approaches to aid in adjustment, well-being, and the EOL experience. This research contributes to growing evidence supporting DT as a meaningful intervention for improving QOL for patients with brain tumors, not just at EOL but earlier in the illness trajectory.

## Appendix B

### GEE linear regression analyses comparing interactions between groups across timepoints

**Table:**

Measure	β	SE	95% CI	*p*
**FACT-Br (Physical WB^a^)**				
Baseline × T1	0.78	0.80	[−0.80, 2.36]	.332
Baseline × T2	−1.03	1.56	[−4.09, 2.04]	.512
T1 × T2	−1.81	1.10	[−3.97, 0.35]	.100
(Baseline × T1) × Group	0.12	1.15	[−2.13, 2.37]	.918
(Baseline × T2) × Group	−0.00	2.08	[−4.08, 4.07]	.998
(T1 × T2) × Group	−0.12	1.41	[−2.90, 2.65]	.931
**FACT-Br (Social WB^a^)**				
Baseline × T1	0.57	0.90	[−1.19, 2.33]	.525
Baseline × T2	0.43	1.22	[−1.96, 2.82]	.722
T1 × T2	−0.14	0.97	[−2.03, 1.76]	.889
(Baseline × T1) × Group	−0.28	1.12	[−2.48, 1.92]	.805
(Baseline × T2) × Group	−1.30	1.43	[−4.11, 1.50]	.363
(T1 × T2) × Group	−1.02	1.12	[−3.22, 1.18]	.362
**FACT-Br (Emotional WB^a^)**				
Baseline × T1	2.19	0.77	[0.68, 3.69]	.004**
Baseline × T2	1.29	0.65	[0.01, 2.56]	.048*
T1 × T2	−0.90	0.66	[−2.20, 0.39]	.172
(Baseline × T1) × Group	−1.41	1.07	[−3.50, 0.68]	.187
(Baseline × T2) × Group	−1.73	1.19	[−4.06, 0.61]	.147
(T1 × T2) × Group	−0.32	0.98	[−2.25, 1.61]	.745
**FACT-Br (Functional WB^a^)**				
Baseline × T1	−0.15	0.70	[−1.52, 1.22]	.826
Baseline × T2	−0.86	0.95	[−2.72, 1.01]	.367
T1 × T2	−0.70	0.96	[−2.59, 1.18]	.465
(Baseline × T1) × Group	1.39	0.92	[−0.42, 3.20]	.133
(Baseline × T2) × Group	−0.85	1.57	[−3.93, 2.23]	.590
(T1 × T2) × Group	−2.24	1.50	[−5.18, 0.71]	.136
**FACT-Br (Brain cancer)**				
Baseline × T1	4.03	3.54	[−2.91, 10.97]	.255
Baseline × T2	−2.98	5.73	[−14.21, 8.26]	.604
T1 × T2	−7.00	4.08	[−15.00, 1.00]	.086
(Baseline × T1) × Group	−5.38	5.03	[−15.24, 4.47]	.284
(Baseline × T2) × Group	−11.03	7.94	[−26.60, 4.53]	.165
(T1 × T2) × Group	−5.65	6.59	[−18.58, 7.28]	.392
**DADDS**				
Baseline × T1	−6.09	3.19	[−12.35, 0.17]	.057
Baseline × T2	−3.38	2.99	[−9.24, 2.47]	.257
T1 × T2	2.70	1.92	[−1.05, 6.46]	.158
(Baseline × T1) × Group	0.98	4.45	[−7.73, 9.70]	.825
(Baseline × T2) × Group	4.07	4.54	[−4.83, 12.96]	.370
(T1 × T2) × Group	3.08	5.42	[−7.54, 13.71]	.569
**UCLA-3**				
Baseline × T1	−0.84	0.52	[−1.86, 0.17]	.104
Baseline × T2	−0.07	0.46	[−0.97, 0.83]	.878
T1 × T2	0.77	0.54	[−0.29, 1.83]	.155
(Baseline × T1) × Group	0.11	0.58	[−1.03, 1.24]	.850
(Baseline × T2) × Group	0.26	0.55	[−0.81, 1.33]	.630
(T1 × T2) × Group	0.15	0.64	[−1.10, 1.41]	.810
**FACIT-Sp-12 (Total)**				
Baseline × T1	2.51	1.66	[−0.74, 5.76]	.131
Baseline × T2	3.00	2.40	[−1.71, 7.71]	.211
T1 × T2	0.49	1.43	[−2.30, 3.29]	.729
(Baseline × T1) × Group	−1.65	2.15	[−5.87, 2.58]	.445
(Baseline × T2) × Group	−4.48	2.92	[−10.22, 1.25]	.125
(T1 × T2) × Group	−2.84	2.34	[−7.42, 1.74]	.224
**FACIT-Sp-12 (Meaning)**				
Baseline × T1	1.02	0.65	[−0.26, 2.29]	.117
Baseline × T2	0.89	0.76	[−0.60, 2.39]	.242
T1 × T2	−0.13	0.47	[−1.04, 0.79]	.789
(Baseline × T1) × Group	−1.19	0.77	[−2.71, 0.32]	.123
(Baseline × T2) × Group	−2.08	0.89	[−3.81, −0.34]	.019*
(T1 × T2) × Group	−0.88	0.77	[−2.40, 0.63]	.254
**FACIT-Sp-12 (Peace)**				
Baseline × T1	0.91	0.70	[−0.45, 2.28]	.189
Baseline × T2	1.22	1.07	[−0.89, 3.32]	.257
T1 × T2	0.30	0.79	[−1.25, 1.85]	.702
(Baseline × T1) × Group	−0.56	1.07	[−2.65, 1.53]	.600
(Baseline × T2) × Group	−1.89	1.35	[−4.53, 0.75]	.161
(T1 × T2) × Group	−1.33	1.19	[−3.66, 1.00]	.264
**FACIT-Sp-12 (Faith)**				
Baseline × T1	1.05	1.17	[−1.24, 3.34]	.368
Baseline × T2	1.52	0.86	[−0.17, 3.20]	.077
T1 × T2	0.47	0.87	[−1.23, 2.17]	.589
(Baseline × T1) × Group	−1.44	1.64	[−4.64, 1.77]	.380
(Baseline × T2) × Group	−1.92	1.08	[−4.02, 0.19]	.075
(T1 × T2) × Group	−0.48	1.36	[−3.15, 2.19]	.725
**PDI (Total)**				
Baseline × T1	−5.63	2.50	[−10.53, −0.74]	.024*
Baseline × T2	−4.36	2.44	[−9.14, 0.42]	.074
T1 × T2	1.27	1.68	[−2.01, 4.56]	.448
(Baseline × T1) × Group	2.40	3.54	[−4.54, 9.35]	.498
(Baseline × T2) × Group	4.35	4.85	[−5.15, 13.84]	.370
(T1 × T2) × Group	1.95	3.76	[−5.43, 9.32]	.605
**PDI (Existential distress)**				
Baseline × T1	−1.69	0.87	[−3.40, 0.03]	.054
Baseline × T2	−1.59	0.84	[−3.24, 0.05]	.058
T1 × T2	0.09	0.57	[−1.03, 1.21]	.870
(Baseline × T1) × Group	0.62	1.17	[−1.67, 2.92]	.596
(Baseline × T2) × Group	2.06	1.58	[−1.03, 5.14]	.192
(T1 × T2) × Group	1.43	1.27	[−1.05, 3.92]	.257
**PDI (Symptom distress)**				
Baseline × T1	−1.72	0.77	[−3.23, −0.20]	.026*
Baseline × T2	−0.78	0.57	[−1.90, 0.33]	.170
T1 × T2	0.93	0.64	[−0.33, 2.20]	.148
(Baseline × T1) × Group	−0.08	0.99	[−2.01, 1.85]	.932
(Baseline × T2) × Group	−0.16	1.32	[−2.75, 2.42]	.901
(T1 × T2) × Group	−0.08	1.09	[−2.21, 2.05]	.942
**PDI (Social support distress)**				
Baseline × T1	−0.74	0.25	[−1.24, −0.25]	.003**
Baseline × T2	−0.64	0.25	[−1.14, −0.15]	.011*
T1 × T2	0.10	0.15	[−0.19, 0.39]	.504
(Baseline × T1) × Group	1.05	0.52	[0.04, 2.06]	.041*
(Baseline × T2) × Group	0.65	0.34	[−0.02, 1.33]	.057
(T1 × T2) × Group	−0.40	0.45	[−1.27, 0.48]	.377
**PDI (Dependency distress)**				
Baseline × T1	0.10	0.25	[−0.39, 0.58]	.690
Baseline × T2	−0.08	0.24	[−0.54, 0.38]	.743
T1 × T2	−0.18	0.24	[−0.66, 0.30]	.473
(Baseline × T1) × Group	−0.34	0.44	[−1.21, 0.52]	.436
(Baseline × T2) × Group	0.65	0.79	[−0.90, 2.20]	.414
(T1 × T2) × Group	0.99	0.66	[−0.30, 2.28]	.132
**PDI (Peace of mind distress)**				
Baseline × T1	−0.94	0.27	[−1.48, −0.41]	<.001***
Baseline × T2	−0.94	0.36	[−1.64, −0.23]	.009**
T1 × T2	0.01	0.18	[−0.34, 0.36]	.967
(Baseline × T1) × Group	0.91	0.56	[−0.19, 2.00]	.106
(Baseline × T2) × Group	0.62	0.55	[−0.46, 1.70]	.259
(T1 × T2) × Group	−0.28	0.37	[−1.00, 0.44]	.439
**PTGI**				
Baseline × T1	2.44	5.87	[−9.05, 13.94]	.677
Baseline × T2	3.42	4.15	[−4.72, 11.55]	.410
T1 × T2	0.97	4.05	[−6.97, 8.91]	.810
(Baseline × T1) × Group	−1.49	8.17	[−17.49, 14.52]	.856
(Baseline × T2) × Group	−10.76	7.11	[−24.69, 3.17]	.130
(T1 × T2) × Group	−9.27	6.10	[−21.23, 2.68]	.128

*Note*: In group comparisons (i.e., “× Group”), the non-EOL group is the reference point.

a Refers to well-being.

* *p* < .05, ***p* < .01, ****p* < .001.
